# Increased lifelong burden of comorbidities without increased early mortality in hereditary hypophosphatemia: a Danish register study

**DOI:** 10.1093/jbmrpl/ziaf143

**Published:** 2025-09-02

**Authors:** Signe Sparre Beck-Nielsen, Rikke Færgemann Hansen, Ulla Ege Johansen, Angela Williams

**Affiliations:** Centre for Rare Diseases, Department of Pediatrics, Aarhus University Hospital, DK-8200 Aarhus, Denmark; Department of Clinical Medicine, DK-8200 Aarhus University, Aarhus, Denmark; Signum Life Science ApS, DK-2100 Copenhagen, Denmark; Signum Life Science ApS, DK-2100 Copenhagen, Denmark; Kyowa Kirin International, Marlow SL7, United Kingdom

**Keywords:** hereditary hypophosphatemia, X-linked hypophosphatemia, comorbidities, life expectancy, retrospective study, diagnosis of hereditary hypophosphatemia

## Abstract

Hereditary hypophosphatemia (HH) is a rare diseases characterized by excessive renal phosphate wasting. Hereditary hypophosphatemia presents as rickets and osteomalacia in children, and osteomalacia in adults. Previous studies have suggested an increased burden of comorbidities and higher risk of early death in individuals with HH compared with controls. This study investigates the comorbidities in HH throughout life and their association with survival compared with controls. Possible HH cases were initially identified from the Danish National Patient Register (DNPR) using diagnostic codes for rickets or hypophosphatemia between 1971 and 2019, and the diagnosis was verified by the review of medical files. A total of 120 individuals with verified HH (case population) were matched by gender, birth year, and month with 50 controls per individual with HH, totaling 6000 controls randomly selected from the Danish Civil Registration System. Comorbidity data related to HH were retrieved from the DNPR. The burden of investigated comorbidities was significantly higher in individuals with HH than controls, with multiple conditions diagnosed and at an earlier age. The lifelong risk of arthrosis was significantly higher and diagnosed earlier in individuals with HH (*p* < .001). By age 50, 31.9% of individuals with HH received their first diagnosis of arthrosis, compared with 4.4% of controls. The lifelong risk of hearing loss was elevated (*p* < .001), often diagnosed by school age, with a rapid increase by age 60. Additionally, risks of hyperparathyroidism and renal failure were higher (both *p* < .001), along with increased rates of hypertensive disease (*p* < .001) and obesity (*p* = .021), with diagnoses increasing from the third decade of life. However, there was no significant increase in ischemic heart disease risk or overall mortality in HH. Further analysis revealed that comorbidities in HH were not associated with a higher risk of death compared with controls with the same conditions.

## Introduction

Hereditary hypophosphatemia (HH) encompasses a group of rare, lifelong, and debilitating inborn disorders characterized by excessive renal phosphate wasting, which leads to chronic hypophosphatemia.^1–3^ The most common forms of HH include X-linked hypophosphatemia (XLH),^3–6^ and autosomal recessive hypophosphatemic rickets type 1 and 2.^6–8^ These conditions result from mutations in genes that regulate phosphate homeostasis, such as the phosphate-regulating endopeptidase homolog X-linked (*PHEX*), dentin matrix protein 1 (*DMP1*), and ectonucleotide pyrophosphate/phosphodiesterase 1 (*ENPP1*).^6–8^

The clinical signs of HH typically present in early childhood as rickets, impaired growth, and skeletal deformities.^4^^,^^9–11^ Adults with HH suffer from progressive comorbidities, including pain, muscle weakness, stiffness, arthrosis, and spontaneous dental abscesses, that worsen with increasing age.^4^^,^^9^^,^^11–13^ Additionally, HH in adults is associated with hearing difficulties, enthesopathy, muscular dysfunction, hyperparathyroidism, and renal complications, all of which tend to worsen over time.^4^^,^^9^^,^^11^^,^^14^ Studies have highlighted the cumulative burden of the disease, showing that as patients age, complications like joint deformities and chronic pain increasingly impair mobility and overall health.^9^ Given the progressive nature of these symptoms, HH considerably impacts health-related quality of life.^15–19^ As patients age, many face difficulties with daily activities, work, and self-care, with substantial effects on social and mental well-being.^15^ Thus, HH potentially evolves into a multi-systemic lifelong disease, affecting many aspects of an individual’s life, which warrants early intervention and multidisciplinary management to improve health status.^9^^,^^15^

Despite the known clinical comorbidities of HH, the impact of these on overall survival has not been fully elucidated. Previous research has suggested an increased risk of early death in individuals with HH.^5^^,^^20^ However, comprehensive studies comparing lifelong survival between individuals with a confirmed diagnosis of HH and a control population are limited.

We conducted a national Danish register-based study to investigate whether lifelong comorbidities in individuals with HH are associated with shortened survival compared with a matched control population. By using real-world, longitudinal data from the Danish National Patient Register (DNPR) and the Danish Civil Registration System (CRS), this research seeks to provide robust insights into the long-term health outcomes of individuals with HH, thereby informing future clinical management and therapeutic strategies.

**Table 1:** Diagnosis codes for identification of potential cases of HH.

**Existence of code in the DNPR**	**SKS-Code**	**Text in the DNPR (English translation)**	**Criteria**
**2012-**	E83.3A1	Familial hypophosphatemia	Included if *>*1 registration
**1994-**	E83.3	Disorders of phosphorus metabolism (Vitamin D resistant: Osteomalacia + rickets)	Included if *>*1 registration
**1994-**	E83.3A	Hypophosphatemia	Included if *>*1 registration
**1994-**	E83.3B	Vitamin D-resistant rickets	Included if *>*1 registration
**1977-1993**	273.40	Familial hypophosphatemia	Included if *>*1 registration

Abbreviations: DNPR, Danish National Patient Register; SKS, Danish Health Service Classification System (Sundhedsvæsenets Klassifikations System).

## Materials and methods

### Study design

This was a nationwide, register-based case-control study using data from Denmark. It included cases of patients with a HH diagnosis confirmed after review of medical files and an age- and gender-matched control population. Diagnosis and comorbidities of cases and controls were collected from 1977 to 2019.

### Case population identification

The case population refers to individuals with confirmed HH through a review of medical files, who were initially identified using diagnosis codes from the DNPR through an application to the Danish Health Data Agency. The DNPR contains individual-level data on all hospitalizations (dates of admission, discharge, and diagnosis) in Denmark since January 1977, including outpatient treatments since 1995. From 1971 to 1993, diagnoses were classified according to the World Health Organization (WHO) International Classification of Diseases, Eighth Revision (ICD-8). Since 1994, the WHO ICD-10 classification system has been used. Possible cases were retrieved from the DNPR for the period from 1977 to December 31, 2019, based on five diagnosis codes related to hereditary rickets or hypophosphatemia: E83.3A1, E83.3, E83.3A, E83.3B, and 273.40 (Table 1). The dataset also included individuals who had died prior to the data collection period, identified as possible cases.

From 1977 to 1993, a specific code for HH was identifiable in the DNPR using the ICD-8 code 273.40. However, with the transition to ICD-10 in 1994, a specific code for HH was no longer available, and only broader diagnostic codes related to phosphate metabolism disorders, hypophosphatemia, and vitamin D-resistant rickets were used. A specific ICD-10 subcode for HH (E83.3A1) was reintroduced in 2012 (Table 1). All potential cases identified through registry data underwent a comprehensive medical file review conducted by SBN to confirm the diagnosis of HH and ensure cohort specificity, throughout the study period.

### Data extraction and verification

**Figure 1:**
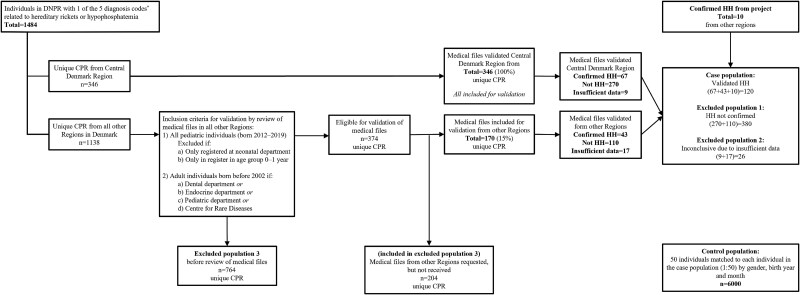
Identification of the case population and matched control population. CPR, personal identification number; DNPR, Danish National Patient Register; HH, hereditary hypophosphatemia. *E83.3A1 or E83.3 or E83.3A or E83.3B or 273.40.

A data extract was obtained from the DNPR, based on five diagnosis codes, which included 3043 contacts with the healthcare system corresponding to 1484 unique individuals (Figure 1). These codes captured both specific HH diagnosis codes (ICD-8: 273.40 and ICD-10: E83.3A1) and broader categories potentially associated with HH. The extract contained the personal identification number (CPR), the code for the responsible hospital, the hospital department, and the date of diagnosis code assignment; however, the specific diagnosis code assigned for the individual could not be obtained. For all individuals identified within the Central Jutland Region, a review of electronic medical files was performed on all available files accessible via the electronic patient medical files system to confirm the diagnosis of HH. Additionally, electronic medical file reviews were conducted in the other four regions of Denmark, but only for a selected portion of the Danish CRS population extracted by the national Danish Health Data Authority. While it was originally planned to identify possible cases with HH across the remaining four regions of Denmark based on the diagnosis codes assigned for hospital admission in the Central Jutland Region, these diagnosis codes were unfortunately not received as a part of the data extract and could not be subsequently transferred. Thus, selection criteria were established for requesting medical files from regions other than Central Jutland, focusing on cases where a diagnosis of HH was considered likely.

### Selection criteria for requesting medical files from other regions

The selection criteria included medical files from all pediatric patients (<18 yr old) by December 31, 2019, excluding cases with only neonatal department records and/or with only 1 contact with 1 of the 5 diagnosis codes assigned before the age of 1 yr; and medical files for adults (18+ yr) with 3 or more contacts by December 31, 2019, including at least 1 contact at the Department of Dental diseases, Department of Endocrinology, Pediatric Department, or Centre for Rare Diseases (Figure 1). Cases with only contacts in departments considered to be unrelated to HH, such as geriatrics, gastrointestinal, nephrology, emergency, hematology, or oncology, were most likely assigned a code of hypophosphatemia due to diseases/conditions other than HH and were therefore excluded. These individuals were then classified as excluded population 3.

Medical files eligible for validation were then requested from the relevant departments (dental diseases, endocrinology, pediatric department, Centre for Rare Diseases, and nephrology), as these were the most likely to follow patients with HH. Individuals from whom medical files from other Regions were requested but not received were also classified as excluded population 3.

### Diagnosis criteria for confirming a diagnosis of HH

**Figure 2:**
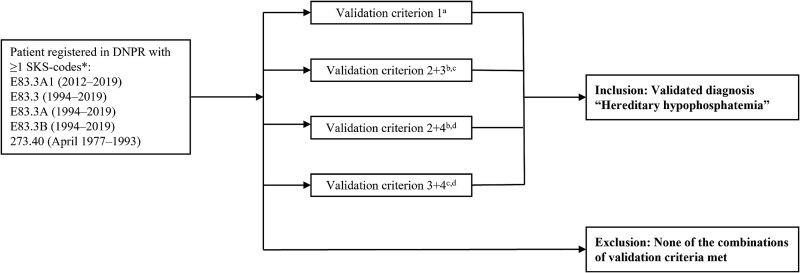
Criteria for verification of HH. DNPR, Danish National Patient Register; HH, hereditary hypophosphatemia; SKS, Danish Health Service Classification System (Sundhedsvæsenets Klassifikations System). *SKS-codes: E83.3A1 familial hypophosphatemia (2012-) or E83.3 disorders of phosphorus metabolism (vitamin D resistant: Osteomalacia + rickets) (1994-) or E83.3A Hypophosphatemia (1994-) or E83.3B vitamin D-resistant rickets (1994-) or 273.40 Hypophosphatemia familiaris (1977-1993). ^a^Criterion 1: verification of XLH by identifying a PHEX mutation or other HH caused by mutations in *DMP1*, *FGF23*, *ENPP1*, or *FAM20C*; ^b^Criterion 2: evidence of X-linked inheritance or first-degree relatives diagnosed with HH; ^c^Criterion 3: history of rickets or spontaneous dental abscesses; ^d^Criterion 4: persistent hypophosphatemia (observed on more than 1 occasion) without the presence of other concurrent diseases, such as alcoholism, severe current illness, medically induced hypophosphatemia by intravenous iron administration, or metabolic bone disease of prematurity.

The criteria for confirming a diagnosis of HH were based on several factors. An overview of these criteria is shown in Figure 2. This validation step was particularly important to distinguish HH from other causes of phosphate metabolism disorders. These included verification of XLH by identifying a *PHEX* mutation or other HH caused by mutations in *DMP1*, *FGF23*, *ENPP1*, or *FAM20C* (criterion 1)*.* A positive diagnosis could also be supported by evidence of X-linked inheritance or first-degree relatives diagnosed with HH (criterion 2). Additionally, a history of rickets or spontaneous dental abscesses (criterion 3), as well as persistent hypophosphatemia (observed on more than 1 occasion) without the presence of other concurrent diseases, such as alcoholism, severe illness, medically induced hypophosphatemia by intravenous iron administration, or metabolic bone disease of prematurity (criterion 4). Hereditary hypophosphatemia was confirmed, if the patient met the following criteria: 1 or (2 + 3) or (2 + 4) or (3 + 4) (Figure 2). Verification of HH based on review of medical files was performed by SBN, except for two hospital departments in Capital Region of Denmark, where the verification was conducted by the treating doctors based on the above listed medical file review criteria. Patients included in the CRS population extract who were excluded during the review of medical files due to a non-confirmed diagnosis of HH were classified as excluded population 1, while those excluded due to an inconclusive diagnosis or insufficient data available, were classified as excluded population 2. In addition, 10 patients with verified HH, identified from an ongoing clinical study but not being retrieved from the search in DNPR using relevant diagnosis codes, were included in the case population (Figure 1).

### Control population

A control population was established by randomly selecting 50 individuals, matched to each patient in the case population (50:1) based on gender, birth year, and month, using the Danish CRS (Figure 1).

To limit the risk of misclassification bias of the control population, case-individuals were not included in the control population. Additionally, control individuals were not first- or second-degree relatives to any of the case-individuals. Individuals in excluded population 3 and 2 were also excluded from the control population.

### Comorbidities

The prespecified comorbidities with a possible relation to HH were selected based on those described in the literature and clinical experience (the list of prespecified comorbidities is provided in the Supplementary Material). Data on comorbidities were extracted from the DNPR, and when an individual was assigned with a diagnosis code, this indicated that the individual was ever diagnosed with this condition.

The comorbidities reported in Table 2 were selected from the prespecified list of comorbidities based on the most frequently registered comorbidities in the case population.

**Table 2:** Lifelong comorbidities and procedure registrations (event) in case-population with HH compared with control population.

**Event**	**Case population (*N* = 120)**	**Control population (*N* = 6000)**	**Difference between the number of individuals assigned with the event between the case and the control population (*p*-value)**	**Risk of experiencing the event in individuals with HH (RR)**
	**Individuals registered at least once with the event (%)**	**Individuals registered at least once with the event (%)**		
**Arthrosis (hip, knee, unspecified)**	23.3	5.6	<.001	4.2
**Hearing loss (excluding congenital)**	16.7	4.0	<.001	4.1
**Hearing aid**	10.8-13.3	2.7	<.001	4.0-4.9
**Hyperparathyroidism**	10.8-13.3	0.3	<.001	34.2–43.0
**Hypertensive disease**	17.5	7.3	<.001	2.4
**Obesity**	13.3-15.8	7.3	.021	1.8–2.2
**Renal failure (acute, chronic, unspecified)**	5.0	0.8–0.9	<.001	5.7–6.0
**Ischemic heart disease**	5.0	3.2–3.3	.406	1.6

RR, relative risk; HH, hereditary hypophosphatemia.

Some data points are presented as ranges due to previously published levels in the child or adult population that were below the limit for discretion.

### Statistical analysis

Descriptive analyses were performed to characterize the distribution of gender and age at first registered diagnosis of HH.

The likelihood of prespecified comorbidities was assessed for cases compared with controls. The percentage of cases and controls registered with at least one diagnosis of arthrosis, hearing loss, hyperparathyroidism, hypertensive disease, obesity, renal failure, and ischemic heart disease was calculated. The percentage of individuals registered to receive hearing aids was also determined. A relative risk (RR) was determined based on the percentage in each of the groups. Chi-squared tests were used to determine if there were statistically significant differences (*p* < .05) between the occurrence of registered events among cases compared with controls.

Kaplan–Meier (KM) estimates were used to investigate the marginal probability of the above prespecified comorbidities at any given age. In the KM estimates, each individual was censored at their age at the end of follow-up if they had not experienced the event by that time. The KM *p*-values were derived using a log-rank test, which compared the likelihood of an event occurring over time between the case population and the control population. A Cox regression was used to compare the age at which comorbidities occurred across 10-yr age intervals in the KM analysis between cases and controls.

The age at death (median [range], years) was calculated for all cases and controls who died on or before December 31, 2019. Eventual differences in age at death for cases compared with controls were analyzed using linear regression, adjusting for birth year and gender. The populations were tested for normal distribution. We calculated the number and percentages of cases with at least one diagnosis of comorbidity (see Supplementary Material), and controls with at least one of the same comorbidities as registered in the cases who died. Logistic regression was performed to analyze the probability of death among cases with comorbidities compared with controls registered with the same comorbidities. From the logistic regression the odds ratio in combination with a 95% CI was calculated to quantify the strength and direction of an association. The analysis was performed adjusted for birth year and gender.

To assess whether the applied inclusion and exclusion criteria were appropriate or may have introduced selection bias, a dropout analysis was performed. The risk of death in the excluded populations 1, 2, and 3 was compared with the risk of death in the control population to determine if the excluded groups had different mortality risks.

All analyses were conducted using the statistical program R studio (R version 4.4.1), via the authority’s secure server system at Statistics Denmark.

Survival curves were smoothed using generalized additive models (GAMs) with integrated smoothness estimation, applied using penalized regression splines from the Mixed GAM Computational Vehicle (mgcv) package in R. This smoothing approach was applied solely for visualization purposes to prevent disclosure of the precise time of events for individual participants, thereby ensuring data confidentiality. Importantly, all statistical analyses and calculations were performed using the original, unmodeled data; only the visual representations of probabilities are based on model-predicted values.

## Results

**Figure 3:**
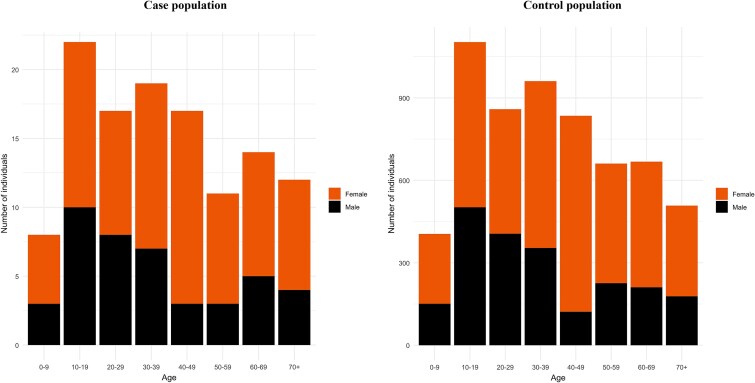
Age distribution of the case and control populations, stratified by gender, at December 31, 2019. In the event of death, age at death was used. Each individual was counted once.

In this study, 120 individuals diagnosed with HH were included in addition to 6000 controls matched by gender, birth year, and month (1:50 ratio). Females comprised 64.2% and males 35.8% in each group (Figure 3).

### Presence of comorbidities

**Figure 4:**
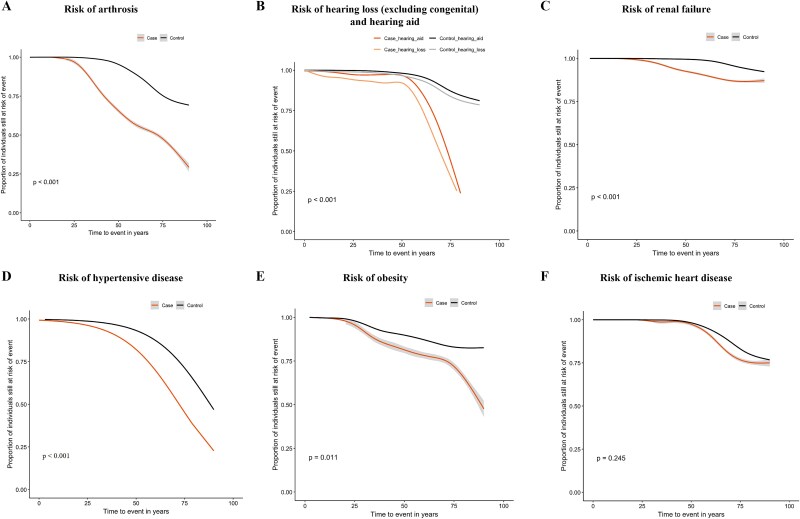
Kaplan–Meier analysis: individuals with HH experience earlier diagnoses of arthrosis, hearing loss (and hearing aid use), hypertensive disease, renal failure, obesity, and ischemic heart disease compared with matched controls. The curves for the cases were smoothened to maintain the discretion level, and the gray shading indicates the possible interval for the smoothened curve.

Individuals with HH face significantly higher lifelong risks of developing pre-specified comorbidities compared with the control group (Table 2). Specifically, 28/120 (23.3%) of individuals with HH developed arthrosis, compared with at least 334/6000 (5.6%) of controls (*p* < .001) (Table 2), with the diagnoses occurring earlier in life (*p* < .001) and being identified from the second decade of life in individuals with HH compared with a later diagnosis among controls according to KM estimates (Figure 4A). By the age of 50 yr, 31.9% of individuals with HH had their first diagnosis of arthrosis, compared with 4.4% of controls.

Likewise, the lifelong risk of hearing loss was significantly higher in the HH group, affecting 20/120 (16.7%) of individuals compared with 241/6000 (4.0%) of controls (*p* < .001) (Table 2). Furthermore, a tendency was observed for an earlier diagnosis of hearing loss in individuals with HH, with 4.0% of cases being identified by school age (0-10 yr) compared with 1.0% in the control group (*p* = .233), in addition to age group 30-40 yr, where 7% of cases had a significantly earlier diagnosis of hearing loss compared with only 1% of controls (*p* = .007). By age 60, there was a rapid increase in the diagnosis of hearing loss among individuals with HH (Figure 4B). In line with these findings, the lifelong risk of being registered to receive a hearing aid was also significantly higher in the HH group. Specifically, >12/120 (10.8%-13.3%) of individuals with HH were registered with a hearing aid at least once compared with only 163/6000 (2.7%) in the control population (*p* < .001) (Table 2). Thus, hearing loss and registration to receive hearing aids occurred more often and at a younger age in individuals with HH (*p* < .001) (Figure 4B).

The HH group showed significantly higher lifelong risks for hyperparathyroidism (>12/120; [10.8%-13.3%] vs 19/6000 [0.3%], *p* < .001) and renal failure (6/120 [5.0%] vs >49/6000 [0.8%-0.9%], *p* < .001) (Table 2). Furthermore, renal failure was diagnosed at an earlier age in individuals with HH (*p* = .026), and by age 40, there was a rapid increase in the diagnosis of renal failure among individuals with HH (Figure 4C).

Hypertensive diseases and obesity were also more prevalent in individuals with HH, with hypertensive disease affecting 21/120 (17.5%) of individuals with HH compared with 436/6000 (7.3%) of controls (*p* < .001), and obesity present in >15/120 (13.3%-15.8%) of the HH group vs 439/6000 (7.3%) of controls (*p* = .021) (Table 2). Both conditions were diagnosed at an earlier age (hypertensive disease: *p* < .001, Figure 4D; obesity: *p* = .011, Figure 4E), and both were increasingly diagnosed from the third decade of life onwards compared with controls.

The lifelong risk of being diagnosed with ischemic heart disease was not increased in individuals with HH compared with controls (5.0% vs 3.2%-3.3%, *p* = .406) (Table 2). Consistent with these findings, the cumulative incidence of ischemic heart disease over time was similar between the case population and the control population (*p* = .245), despite an apparent difference in the 60-70 age group, that was not statistically significant (Figure 4F).

### Mortality

**Figure 5:**
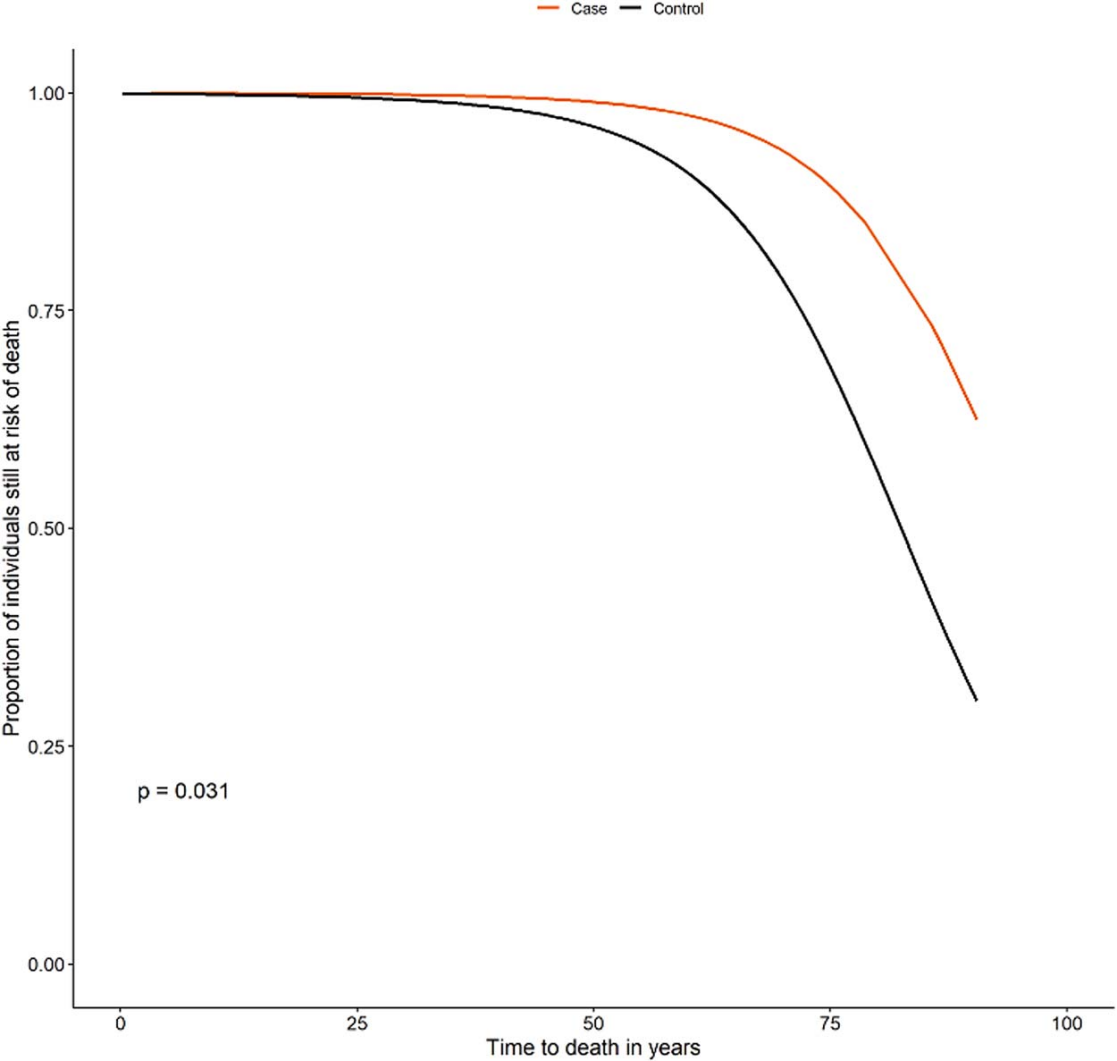
Kaplan–Meier analysis of the lifelong risk of death until December 31, 2019.

Risk of death was not elevated in individuals with HH compared with the control group, where the *p*-value of .031, Figure 5, indicates a higher lifelong risk of death in the control group compared to individuals with HH. Despite the increased burden of comorbidities, the survival analysis showed no statistically significant difference in age at death between individuals with HH who had at least one comorbidity and the control group with at least one of the same comorbidities (Table 3). The median age at death was at age 69 for individuals with HH vs age 68 for the control group, and adjusted estimates, accounting for birth year and gender, showed a non-significant deviation of 2.94 yr of age at death (95% CI: −5.7 to 11.57, *p* = .502) as shown in Table 3. Furthermore, the presence of comorbidities in the case population was not associated with an increased odds ratio of death compared with the control population with at least one of the same comorbidities (odds ratio = 0.38, *p* = .149) (Table 4).

**Table 3:** Age at the time of death for the case population with at least 1 comorbidity^a^ and for the control population with at least 1 matching comorbidity, who died up to and including December 31, 2019.

	**Number of individuals who had at least 1 comorbidity** ^**a**^			
	**Case population** **(*N* = 79)**	**Control population** **(*N* = 1195)**	**Difference in age at death between groups analyzed by linear regression** ^**b**^	
	**Median age** **years (range)**	**Median age** **years (range)**	**Difference in median age (95% CI)**	***p*-value**
**Age at death in individuals with at least one comorbidity**	69 (48; 70)	68 (13; 89)	2.94 (−5.70 to 11.57)	.502

a Comorbidities in cases who died were at least 1 of the following conditions: spinal stenosis, dwarfism, depressive episodes, arthrosis, hearing loss (excluding congenital hearing loss), tinnitus, ischemic heart disease, diseases of the pulp and periapical tissues, renal failure, hypertensive disease, and hyperparathyroidism.

b Adjusted to birth year and gender.

**Table 4:** Risk of death up to and including December 31, 2019, calculated using the odds ratio for the case population with at least 1 comorbidity, and for the control population with at least 1 matching comorbidity.

	**Number of individuals who had at least 1 comorbidity** ^**a**^		**Difference in risk of death between groups analyzed by logistic regression** ^**b**^	
	**Case population (*N* = 79)**	**Control population (*N* = 1195)**	**Adjusted OR (95% CI)**	***p* -value**
	***n* (%)**	***n* (%)**		
**Number of individuals with at least 1 comorbidity, who died**	<5 (−)	164 (13.7)	0.38 (0.10; 1.41)	.149

OR, odds ratio.

a Comorbidities were defined as having at least 1 of the following conditions: spinal stenosis, dwarfism, depressive episodes, arthrosis, hearing loss (excluding congenital hearing loss), tinnitus, ischemic heart disease, diseases of the pulp and periapical tissues, renal failure, hypertensive disease, and hyperparathyroidism.

b Adjusted to birth year and gender.

### Dropout analysis

**Figure 6:**
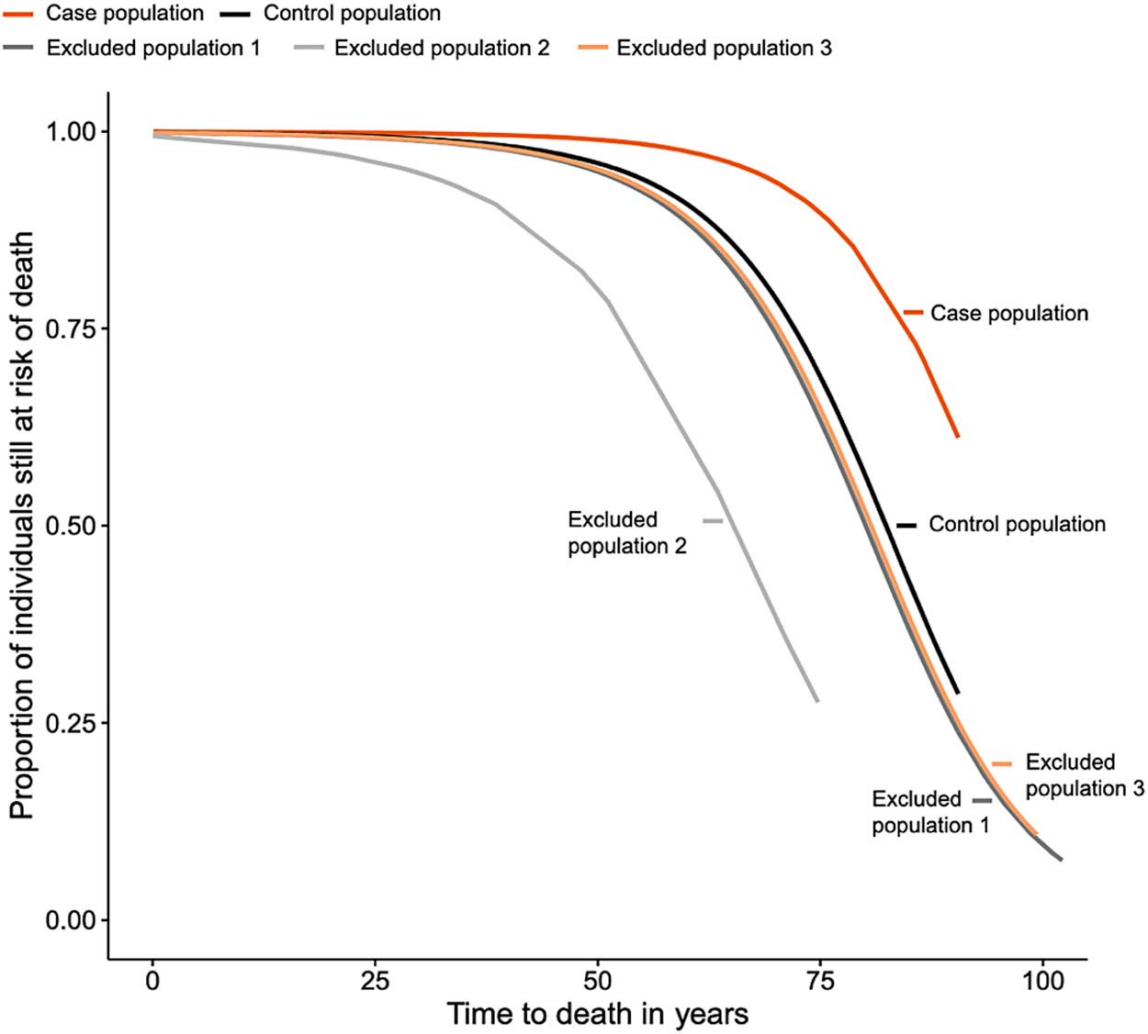
A Kaplan–Meier analysis of the risk of death until December 31, 2019 across all populations. CSR, Civil Registration System. *Case population*, *n* = 120: patients included in the CRS population extract and included at review of medical files, due to a confirmed diagnosis of hereditary hypophosphatemia (case population). *Excluded population 1*, *n* = 380: patients included in the CRS population extract and excluded at review of medical files, due to a non-confirmed diagnosis of hereditary hypophosphatemia. *Excluded population 2*, *n* = 26: patients included in the CSR population extract and excluded at review of medical files, due to inconclusive diagnosis. *Excluded population 3*, *n* = 968: patients included in the CRS population extract and excluded before review of medical files.

In the case population (*n* = 120), which included patients from the CSR with a confirmed diagnosis of HH after review of medical files, 0.8%-3.3% of cases died within the time of follow-up December 31, 2019. This data point is presented as a range because the number of deaths was below the minimum reporting threshold. In excluded population 1 (*n* = 380), consisting of patients who were included in the CSR population extract but excluded at the review of medical files due to a non-confirmed diagnosis of HH, 25.0% of patients died. In excluded population 2 (*n* = 26), consisting of patients with an inconclusive diagnosis of HH, 19.2% of patients died. Lastly, in excluded population 3 (*n* = 968), consisting of patients who were excluded before the review of medical files, 47.1% of patients died. The odds ratio (95% CI) for death were 3.4 (2.4-4.8), 5.5 (1.5-20.5), and 4.2 (1.2-1.8) for excluded populations 1, 2, and 3, respectively, when compared with the control population. In addition, the curves showing the risk of death for the population excluded before and at the medical file review (excluded population 1 and 3, respectively) were almost overlapping with the control population (Figure 6).

Of the 380 individuals, where HH was not verified (excluded population 1), a total of 190 (54%) suffered from other conditions often being linked to early death, such as alcoholism (*n* = 51, 13%), cancer of any kind (*n* = 36, 10%), anorexia or other nutritional deprivation (n = 31, 8%), sepsis or severe infection (*n* = 24, 6%), prematurity (*n* = 20, 5%), inflammatory bowel disease (*n* = 17, 4%), ketoacidosis (*n* = 6, 1%) or had received iron infusions (*n* = 5, 1%).

## Discussion

This retrospective analysis is the first nationwide, population-based epidemiology study to examine the association between comorbidities and mortality in individuals with HH. Utilizing a comprehensive register derived dataset from the DNPR in Denmark, our findings demonstrate that individuals with HH are at significantly higher risk for a range of comorbidities than age and gender-matched controls. However, despite the elevated prevalence of comorbidities and their earlier diagnoses compared with matched controls, these comorbidities do not appear to translate into a reduced lifespan, as the overall survival was not shortened in people with HH compared with controls. Additionally, the presence of comorbidities in the case population was not associated with an increased risk of death when compared with the control population with the matching comorbidities as registered in the case population.

This study demonstrates a significantly increased risk and earlier onset of comorbidities of arthrosis, hearing loss, hyperparathyroidism, renal failure, hypertensive disease, and obesity among individuals with HH compared with controls. These findings align with previous reports that suggest an increased presence of musculoskeletal and metabolic complications in HH.^4^^,^^9^^,^^11^ The earlier diagnosis of arthrosis in HH, often by the second decade of life, corroborates previous studies highlighting the musculoskeletal challenges faced by individuals with HH.^3^^,^^9–12^ The significantly higher prevalence and earlier diagnosis of hearing loss in the HH group are consistent with previous studies.^9^^,^^21^ While malformation and impaired mineralization of the bones of the inner ear have been described in mouse models, their direct relevance to human cases remains unclear.^22^^,^^23^ Furthermore, our study showed that individuals with HH face a significantly higher likelihood of requiring a hearing aid as part of necessary auditory intervention. Hyperparathyroidism and renal failure were also more common in individuals with HH and, in some but not all studies, have been linked to high phosphate dosing regimens.^9^^,^^24–29^ The increased incidence of hypertensive disease and obesity call for awareness in the clinical management of HH,^3^ as these conditions are known to exacerbate cardiovascular risk, although, this study did not find a statistically significant increase in ischemic heart disease among the HH population.

These findings challenge earlier assumptions. While UK and South Korean studies reported reduced survival, our data show no mortality difference.^5^^,^^20^ A population-based cohort study using the large primary care, UK Clinical Practice Research Datalink GOLD dataset in the UK, analyzed data from 1995 to 2016 on XLH. Potential XLH cases were identified using diagnostic codes related to rickets or osteomalacia and graded based on clinical records, laboratory results (eg, low phosphate levels) and treatment history (eg, long-term activated vitamin D). Each case was matched by age, gender, and by the same general practitioner (GP) with up to four controls who lacked XLH-related codes. Trends in prevalence over the study period were estimated (stratified by age), and survival among cases was compared with that of controls. The analysis revealed a reduced survival[among 122 at least possible cases of XLH, where 27 were classified as highly likely, 37 likely, and 58 possible cases of XLH, relative to non-XLH controls. The average age at death was approximately 8 yr younger in cases relative to age-matched and sex-matched controls (age 64 vs 72.5). The study found significantly increased mortality in those with possible XLH, with a hazard ratio (HR) of 2.93 (95% CI, 1.24-6.91). For those with likely or highly likely XLH, the mortality risk was even higher, with an HR of 6.65 (95% CI, 1.44-30.72).^5^ Similarly, a nationwide study in South Korea on chronic idiopathic hypophosphatemia (CIH) utilized the Korean Health Insurance Review and Assessment claims database to evaluate the incidence of CIH diagnoses from 2003 to 2018. Potential CIH cases were identified using diagnostic codes for phosphorus metabolism disorders or rare intractable disease (RID) codes, requiring at least 2 relevant diagnoses in the index year or 1 RID code, along with prescriptions for active vitamin D within 6 mo. Exclusions included conditions like Fanconi syndrome, hypoparathyroidism, or chronic kidney disease to eliminate secondary causes of hypophosphatemia. A total of 154 cases with non-secondary and non-renal hypophosphatemia were matched by age, sex, and index year controls with a 1:10 ratio to assess complications and mortality. This analysis found that hypophosphatemic patients had a higher risk of mortality compared to age-matched and sex-matched controls (adjusted HR [aHR], 3.26; 95% CI, 1.83-5.81).^20^ In contrast, our study revealed no significant difference in the median age at death, with individuals with HH having a median age of 69 yr compared with 68 yr in the control population.

Several factors could explain the differences in mortality outcomes between these studies. Of consideration, the baseline characteristics across studies varied reflecting different demographic and health profiles between the populations in the UK, South Korea, and Denmark. Additionally, variations in healthcare systems, access to medical care, and socioeconomic factors between these countries could influence the overall health and survival of individuals with HH.^30^ Although these countries have universal healthcare systems, different healthcare dynamics can affect the management of chronic conditions, such as HH. In Denmark, for example, access to GPs and hospitals as well as surgical procedures is free of charge. There is a public medicine subsidy, and various public transfer income options are available, including early retirement benefits, rehabilitation, flexible jobs, and sick pay. These social safety benefits may have an impact on the management of chronic diseases and ultimately the health of individuals living in Denmark. Moreover, genetic backgrounds, environmental factors, lifestyle, diet, and other health-related behaviors specific to each population could influence disease progression and outcomes. Variations in the control group composition, matching criteria, or data collection methods could contribute to the observed differences in mortality trends. In addition, the methods used in the above-mentioned studies may unintendedly include some patient groups with hypophosphatemia, as a result of alcoholism, cancer, anorexia or other nutritional deprivation, sepsis or severe infection, prematurity, inflammatory bowel diseases, ketoacidosis, or having received iron infusions, all having a higher risk of early death compared with the control population. Being included as suspected HH, these patients may likely confound the results, underlining the importance of verifying the HH diagnosis before inclusion. Therefore, these results should be interpreted with caution and contextual factors must also be considered when comparing findings across different regions and time periods. Future research should aim to further explore these factors to better understand the long-term outcomes of individuals with validated HH across different settings.

Given the present study, it is recommended to perform screening for hearing loss starting at school age in children with HH, with efforts intensified for middle-aged individuals. In addition, implementing lifestyle interventions targeting obesity and hypertensive disease requires increased attention, starting from the transition to adulthood and continuing thereafter. In line with previous findings, comprehensive, multidisciplinary clinical evaluation, and continuous monitoring of severity and treatment efficacy are crucial for maintaining quality of life and potentially reducing the impact of these conditions on morbidity and mortality.^31^

This study has several strengths, including the use of extensive and longitudinal data from the DNPR, which offered detailed information on hospitalizations and outpatient treatments from 1977 to 2019. The reliability of the HH diagnosis was enhanced by a thorough review of medical records. The selection criteria for requesting medical files for review was considered robust, as the risk for dying was very similar in the two populations being excluded before and after review (population 3 and 1, respectively). Furthermore, the control population was matched to the case population based on gender, birth year, and month, with stringent criteria applied to minimize misclassification bias. However, there are limitations to consider. Both the case and control populations were subject to right censoring, which may limit the accuracy of the mortality outcomes. The hospital policy of deleting medical records after 10 yr for patients who did not continue to attend follow-up appointments, coupled with the lack of digitization of many paper records from several years ago, resulted in incomplete data, particularly regarding the long-term outcomes. Furthermore, as with any registry-based study, some biases are likely to be introduced. For instance, surveillance bias is a concern because the DNPR only captures diagnoses related to hospital visits, and not visits to the GP. Patients with rare diseases like HH are more likely to be enrolled in the hospital system, thus more likely of being referred to specialized care, where diagnoses, such as osteoarthritis, are more likely to be identified and coded, compared to patients only seen by their GP. This helps reduce the likelihood of comorbidities being underreported. Consequently, conditions that do not require hospital care or pre-surgical evaluations might be underreported, potentially leading to an overrepresentation of certain comorbidities, particularly in the case population. Some diagnosis codes were not actively in use in DNPR with only a few registrations as for example tobacco use (Z72.0) and were thus omitted from analysis. The retrospective design and dataset based on historically assigned diagnosis codes introduced inherent challenges, which may have led to an underestimation of the true prevalence of comorbidities and their impact on survival.

## Conclusion

Our study reveals a significantly higher burden of comorbidities in individuals with HH, with earlier diagnoses compared with the controls. Notably, despite this increased comorbidity burden, our findings indicate that the presence of comorbidities does not correlate with a higher risk of death. Given the progressive and accumulative burden of disease in HH as individuals age, there is a need to prioritize the provision of necessary care to enhance their health-related quality of life and appropriately address their comorbidities. Future research should focus on providing a more detailed understanding of the long-term health outcomes in this population, allowing for the development of more targeted therapeutic strategies.

## Supplementary Material

Danish_Registry_Supplementary_material_JBMR_10Mar2025_ziaf143

## Data Availability

Signum Life Science ApS analyzed data relating to the populations, demography, diagnosis, and treatment using available data in Danish registries. The study was conducted on pseudo-anonymized data and Signum works in accordance with the General Data Protection Regulation (EU) 2016/679 of 27/04/2016 and the Danish Act of Processing of Personal Data Act no. LBK 289 of 08/03/2024.
