# Supplementary material for: Increased lifelong burden of comorbidities without increased early mortality in hereditary hypophosphatemia: a Danish register study
Source: JBMR Plus. 2025 Sep 2;9(10):ziaf143. doi: 10.1093/jbmrpl/ziaf143 (PMC12461693; doi:10.1093/jbmrpl/ziaf143)
Supplement: Danish_Registry_Supplementary_material_JBMR_10Mar2025_ziaf143 [file danish_registry_supplementary_material_jbmr_10mar2025_ziaf143.pdf]

**SUPPLEMENTARY MATERIAL:**

List of diagnosis codes describing possible comorbidities in HH:

Q750: Craniosynostosis

Q070: Chiari I malformation

M480: Spinal stenosis

M257: Osteophytes (anywhere)

E343: Dwarfism

M159/M16-M169/M17-M179/M19-M199: Arthrosis: M16 hip; M17 knee; M19 unspecified

M460/M76-769/M77-M779: Enthesopathies: M460 spine; M76 leg; M77 other enthesopathies; M773 heel

H80-H809: Otosclerosis

H90-H908 (excl. H905E)/H91-H919: Hearing loss, excl. congenital hearing loss (H905E)

H931: Tinnitus

I10-I15: Hypertensive disease

I20-I25: Ischemic heart disease

E65-E66: Obesity

N17-N179/N18-N189/N19-199 Renal failure: N17 acute; N18 chronic; N19 unspecified

N288J: Nephrocalcinosis

N200(A-Z): Kidney stones

I109: Hypertension

E21-E213 (excluding E210B): Hyperparathyroidism

O65-O654: Obstructed labour due to maternal pelvic abnormality (excluding abnormal maternal pelvic organs)

K04-K049: Diseases of pulp and periapical tissues

M218F: Coxa vara

M218H: Coxa valga

M218E: Genu varum

M218C: Genu valgum

F32-329/F33-339: Depressive episode F32/Recurrent F33

F43-439: Reaction to severe stress, and adjustment disorders
